# Supplementary material for: Feasibility and acceptability of early infant screening for sickle cell disease in Lagos, Nigeria—A pilot study
Source: PLoS One. 2020 Dec 3;15(12):e0242861. doi: 10.1371/journal.pone.0242861 (PMC7714115; doi:10.1371/journal.pone.0242861)
Supplement: S1 Appendix — (PDF) [file pone.0242861.s001.pdf]

# INFORMED CONSENT DOCUMENT

**Research IDNO:** ____________________________________________

**Title of the research:** Feasibility and Acceptability of Early Infant Screening for Sickle Cell Disease in Nigeria--------A Pilot Study in Somolu Local Government Area in Lagos State.

**Name(s) and affiliation(s) of researcher(s) of applicant(s):** This research study is being conducted by a team of researcher led by Dr E.O. Oluwole of the Department of Community Health and Primary care, College of Medicine University of Lagos.

**Sponsor(s) of research:** This research was supported by the Fogarty International Center of the National Institutes of Health under Award Number D43TW010134.

**Purpose(s) of research:** This study aims to pilot an early infant screening program for sickle cell disease (SCD) in Somolu Local Government Area (LGA) of Lagos State to determine the feasibility of a Statewide infants' SCD screening program.

**Procedure of the research, what shall be required of each participant and approximate total number of participants that would be involved in the research:** The research involves mothers and their infants presenting to Primary Health Care centres in Somolu LGA for routine immunization.

If you are willing to participate, the procedure is a very simple and straight forward. It requires you to supply some information; that is an interviewer will ask you a series of questions about SCD from a questionnaire, which you need to answer as truthfully as possible. The purpose of this is to enable us determine the feasibility and acceptability of early infant for SCD screening. After which, a drop of blood sample will be obtained from you and your baby by trained phlebotomist for the rapid kit genotype testing and you will be given the results immediately, for the confirmatory test result, you will receive a call from us.

**Expected duration of research and of participant(s)' involvement:** Participants 'involvement is at the first point of administering a questionnaire, pre-counselling and testing for SCD and at the point of post-counselling and collection of screening result.

**Risk(s):** No harm whatsoever is anticipated concerning any of the respondents.

**Costs to the participants, if any, of joining the research:** Participants will not be required to pay any fees for participating in this research.

**Benefit(s):** The study gives each respondent a unique opportunity to know their genotype and also that of their infants. In addition, for those infants that will be diagnosed of SCD, they will have the opportunity for referrals and early commencement of treatment at SCD clinic in LUTH to prevent disease complications.

**Confidentiality:** All the information provided will be kept strictly confidential, and used for research purpose only. All information collected will be given code numbers and no name will be recorded.

**Voluntariness:** You are assured that your participation in the research is voluntary and you are free not to participate, no punishment will be attached to your decision and you can choose to withdraw from the study at any time.

**Consequences of participants' decision to withdraw from research and procedure for orderly termination of participation:**

This is a day's participation, which will not take more than twenty minutes of your time. However, you are free to refuse to answer the questionnaire or being bled, which implies that you are not interested in the research. Please understand that we will make good effort to comply with your wishes as much as possible.

**Modality of providing treatments and action(s) to be taken in case of injury or adverse event(s):** We do not envisage neither injury nor any form of adverse effects from this research. However, First Aid Box will be made available in case of any injury.

**What happens to research participants and communities when the research is over:**

All the research participants will have the results of their genotype screening and of their infants. All the infants that is positive for SCD following screening will be referred to the SCD clinic in LUTH and adequate follow up of the infants will be made even when the research is over to make sure they are keeping to the appointments and other necessities.

**Statement about sharing of benefits among researchers and whether this includes or exclude research participants:**

There is no financial benefit from this study. The researchers will only benefit from being an author in paper publication(s) that emanates from the research when it is concluded. The research participants can have access to the results of the research if they are interested.

**Any apparent or potential conflict of interest:** None

**In case of any enquiry, please contact the following:**
**Principal Investigator:** …………
Dr. E.O. Oluwole
Department of Community Health and Primary Care
College of Medicine, University of Lagos.
08035673029
eaoluwole@unilag.edu.ng

**The Chairman of the CMUL HREC …**
Prof. S.A. Omilabu
Chairman Health Research and Ethics Committee CMUL

08023119431

## INFORMED CONSENT CERTIFICATE

**Statement of person obtaining informed consent (Research Assistant)**

I have fully explained this research to ____________________________________ and have given sufficient information, including about risks and benefits, to make an informed decision.

Name: ______________________________________________

Phone No: _______________________________

Signature: _______________________________ Date: _____________________

**Statement of person giving consent (Respondent)**

I have read the description of the research or have had it translated into language I understand. I have also talked it over with the doctor to my satisfaction. I understand that my participation is voluntary. I know enough about the purpose, methods, risks and benefits of the research study to judge that I want to take part in it. I understand that I may freely stop being part of this study at any time. I have received a copy of this consent form and additional information sheet to keep for myself.

**Research IDNO: ______________________________________________**

Phone No: _______________________________

Signature: _______________________________ Date: _____________________

PLEASE KEEP A COPY OF THE SIGNED INFORMED CONSENT CERTIFICATE.

**FEASIBILITY AND ACCEPTABILITY OF EARLY INFANT SCREENING FOR SICKLE CELL DISEASE IN NIGERIA--------A PILOT STUDY IN SOMOLU LOCAL GOVERNMENT AREA IN LAGOS STATE.**

**Questionnaire**

## Section A: Socio-demographics characteristics of respondents

(*DK= Don't know)

**Research IDNO: ____________________________________________**

1. Age in years --------------------(last birthday)
2. Marital status (a) Single (b) Married (c) Divorced/separated (d) Widow
3. Highest level of education completed (a) None (b) Primary (c) Secondary (d) Tertiary
4. Age of infant -----------------------------------(weeks) pls indicate
5. Gender of the infant (a) Male (b) Female
6. Birth order of the infant (a) first (b) second (c) third (d) fourth (e) ≥5
7. Did you register for antenatal care (ANC) during pregnancy? (a) Yes (b) No
8. Where pls? (a) Tertiary/Teaching Hospital (b) General Hospital (c) PHC (d) Private Hosp. (e) TBA home
9. If yes, number of antenatal care visits (state the number of times) ---------------------------
10. Employment status: (a) Employed (b) Unemployed

### Section B: Feasibility and Acceptability of Early Infant Screening for Sickle Cell Disease

| S/N | Statements | YES | NO | DON'T KNOW |
|---|---|---|---|---|
| 11. | Would you like to be screened for your genotype now? | | | |
| 12. | Would you like your infant to be screened for genotype now? | | | |
| 13. | If Yes to Q51 or 52; Why do you/child want to be screened? Because **(multiple answers applied)**<br>• I just want to know my genotype and that of my child<br>• I have a child now with SCD<br>• I had a child with SCD before but late<br>• I and my spouse are both carriers of SCD hemoglobin<br>• The screening is free for me and my child<br>• Any other reason(s) ……………………………….. pls. state | | | |
| 14. | If No to Q51 or 52; What are the reasons for declining to be screened? (**multiple answers applied**)<br>• I already know my genotype<br>• I already know my child's genotype<br>• My husband and I are both AA | | | |

| | | | | | |
|---|---|---|---|---|---|
| | • I do not want to know the genotype to avoid being worried<br>• I do not want my child to be pricked/bled<br>• It will take time for the result to be ready<br>• I do not like free test<br>• Any other reason (s), pls state. ----------------------------- | | | | |

15. In your view, how early do you think baby should be screened for genotype? (a) in pregnancy (b) immediately at birth (c) within the first one-month (e) pre-school (f) others ------------------------------

16. If your child is discovered to be SS/SC, will you want to enroll him/her in SCD clinic immediately to prevent complication of the disease? (a) Yes (b) No

17. Considering the benefit of early infant screening for SCD, would you be willing to pay ₦1,700.00 from your pocket to screen your child early for genotype in the future? (a) Yes (b) No (c) Not sure

18. Can you afford to pay ₦1,700.00 from your pocket to screen your child early for genotype?
(a) Yes (b) No (c) Not sure

19. Would you be willing to pay ₦7000.00 for additional test to confirm if your child tested positive for SCD? (a) Yes (b) No (c) Not sure

20. Can you afford to pay ₦7000.00 from your pocket to for additional test to confirm if your child tested positive for SCD? (a) Yes (b) No (c) Not sure

21. What in your view would be the challenges of early infant screening for your child? **(multiple answers applied)**

a. Cost of screening test at ₦1700 (a) Yes (b) No
b. Cost of confirmatory test at ₦7000 (a) Yes (b) No
c. Availability of test facility (a) Yes (b) No
d. Accessibility to test result (a) Yes (b) No
e. Time committed to counselling and testing (a) Yes (b) No
f. Availability/accessibility for follow up care (a) Yes (b) No
g. Fear of knowing the SCD status of the child (a) Yes (b) No
h. Delay in getting the test result (a) Yes (b) No
i. Any other reason pls. state. ---------------------------------------------------------

**Research IDNO: ______________________________________________**

**THANK YOU.**
