# Supplementary material for: Feasibility and acceptability of early infant screening for sickle cell disease in Lagos, Nigeria—A pilot study
Source: PLoS One. 2020 Dec 3;15(12):e0242861. doi: 10.1371/journal.pone.0242861 (PMC7714115; doi:10.1371/journal.pone.0242861)
Supplement: S3 Appendix — (PDF) [file pone.0242861.s003.pdf]

COLLEGE OF MEDICINE, UNIVERSITY OF LAGOS
HEALTH RESEARCH ETHICS COMMITTEE

CMUL HREC Registration Number: HREC/15/04/2015

Office Address: 2nd Floor, Biomedical Engineering Block, College of Medicine, University of Lagos P.M.B. 12003, Lagos, Nigeria

Telephone: 0802 864 2463 E-mail: hrec@cmul.edu.ng Website: cmul.unilag.edu.ng

Chairman:
Prof. Sunday A. Omilabu
B.Sc. (Hons.) (Ife), M.Sc. (Ibadan), Ph.D. (Ibadan)

Vice-Chairman:
Dr. Kolawole S. Oyedeji
B.Sc. (Uniport), M.Sc. (Ife), MHRE (South Africa), Ph.D. (Lagos), AIMLS (Nig.), CPHPM (Ilorin)

29th April, 2019

Re: Feasibility and Acceptability of Early Infant Screening for Sickle Cell Disease In Nigeria-A Pilot Study In A Local Government Area In Lagos State.

CMULHREC Number: CMUL/HREC/03/19/503
Name of Principal Investigators: Dr. Esther Oluwakemi OLUWOLE
Date of receipt of valid application: 7th March, 2019

Date of meeting when final determination of research was made: 20th February, 2019

APPROVAL LETTER

The above named proposal has been adequately reviewed; the protocol and safety guidelines satisfy the conditions of **CMULHREC** policies regarding experiments involving human and or animal participants.

Therefore, the study under its reviewed state is hereby **approved by the Health Research Ethics committee of College of Medicine of the University of Lagos.**

[signature] 03/05/19

PROF. S.A. OMILABU
Name of CMULHREC Chairman

Signature & Date

[signature] 3/5/19

Dr. K. S. Oyedeji
Name of CMULHREC Member

Signature & Date

This approval is given with the investigator's responsibility declaration as attached and that;

i) You will submit in CMULHREC prescribed forms, annual progress report during the course of this study, if it is more than one year and final report as the case may be if less than one year and after completion of the study.

ii) The CMULHREC reserves the right to monitor and review this approval; even after the commencement of your study and inform you of any further changes or amendments that may be required for your compliance.

This approval dates from **29/04/2019 to 28/04/2020.** If there is delay in starting the research, please inform the HREC so that the dates of approval can be adjusted accordingly.

Scanned with CamScanner
