# Supplementary material for: Feasibility and acceptability of early infant screening for sickle cell disease in Lagos, Nigeria—A pilot study
Source: PLoS One. 2020 Dec 3;15(12):e0242861. doi: 10.1371/journal.pone.0242861 (PMC7714115; doi:10.1371/journal.pone.0242861)
Supplement: Attachment — Submitted filename: Response to Reviewers.docx [file pone.0242861.s004.docx]

RESPONSES TO EDITOR AND REVIEWERS’ COMMENTS

Editor Comments:
Your paper addresses and important question of public health importance in Africa, and globally: the screening of Sickle cell disease.
Although the authors have a genuine attempt to address your request question, in its present form, the methodology and quality of the reporting need some major improvement as indicated by both reviewers.
Moreover, more background information on the setting (Nigeria) are needed to put the paper into context.
Population size? SCD incidence and prevalence? General level of formal education? Poverty indices? National health system? Available public health policies and current practices regarding SCD screening; Availability and access to comprehensive services for SCD prevention and care.
Please carefully address point-to-point the editor and reviewers’ comments, as this is expected to improve the quality of your text.

Author’s Comments:

Thank you, sir, for your comments.

The methodology and quality of the reporting has been reviewed with major improvement as indicated by the reviewers. Also, more background information on the setting (Nigeria) have been added to put the paper into context in the method section. (Page 6-7; Lines 134-148)

We have carefully addressed the point-to-point the editor and reviewers’ comments, to improve the quality of the manuscript.

| **Reviewer #1** | **Original comments of the reviewer** | **Reply by the author(s)/ Changes Made** | **Changes done on page number and line number** |
| --- | --- | --- | --- |
| **General** | In general terms, I would advise the authors to refer to the diagnoses as Hb genotypes instead of phenotypes so as not to be confused with clinical phenotypes. | Thank you, Sir, for all the comments.  This has been corrected throughout the manuscript | All pages |
| **Introduction** | There’s need to include in the introduction, the justification for using POCT in newborn screening in preference to other available screening methods especially in resource-poor areas. A brief mention of previous work with HemoTypeSC in Nigeria and other places is also apropos. | These has been included in the introduction. | Pages 4-5  Lines 90-99 |
|  | There are many grammatical and syntax issues and the authors are advised to review the whole manuscript carefully in this regard. Some suggested corrections are outlined below: | Grammatical and syntax issues have been addressed carefully. | All pages |
| **Abstract:** | Line 31, should read “……comprehensive management is not…..” | Corrected | Pg. 2; Line 33 |
|  | Line 39, should read “The haemoglobin genotype of mother-infant pairs…..” | Corrected | Pg. 2; Line 41 |
|  | Line 41, should read “Data were analysed with SPSS version 22”. | Corrected | Pg. 2; Line 43 |
|  | The whole of the introduction needs to be restructured. Currently it just one long paragraph and it does not flow well. Apart from briefly introducing sickle cell disease epidemiology, it should focus on the problems associated with newborn screening and the current methods as mentioned above Line 59 - 62,  “In high income countries where the infrastructure for universal newborn screening, early intervention and comprehensive care exist mortality has reduced from 16% to <1% with less than 1% global disease burden and over 90% of babies born with SCD surviving into adulthood”. It is not clear if the 16 to 1% figure is referring to overall mortality or only in childhood. | DONE accordingly  This statement has been rephrased | Pages 3-6;  Lines 53-128  Pg.4;  Lines 77-80 |
| **Materials and Methods** |  |  |  |
|  | Lines 88 – 90. The sentence “The State has the highest population in Nigeria with twenty local government areas (LGA), and this study took place in one of them which was selected by simple random sampling through ballot” needs to be restructured. | The statement has been restructured | Pg. 7  Line 150-151 |
|  | Lines 127 – 133. Most of the description of HemoTypeSC given here rightly belongs in the introduction. | The description of HemoTypeSC has been moved to the introduction. | Pg. 5  Line 101-108 |
|  | Line144, should read: “…heelprick of each of the infants by trained ….” | Corrected | Pg. 8;  Line 188 |
|  | Line 147, The infants’ blood samples were….” | Corrected | Pg. 9;  Line 191 |
|  | Line 153, should read “…. while the results of the confirmatory test were communicated….” | Corrected | Pg. 9;  Line 201 |
|  | Line157 – 162. This section, starting with “Feasibility.” should be in a new paragraph.  Moreover, it is not clear how the authors arrived at the cut-off figures quoted. Are they from previous studies? If so, the source should be referenced; otherwise their basis should be explained. | This section has been moved to a new paragraph  The cut-off figures were the standard set for the feasibility before the study was conducted. These data shows that the intervention is practical and feasible.  They were not quoted from previous study | Pg. 9;  Lines 207-212 |
| **Results:** | Line 174, should read, “Socio-demographic characteristics of mother-infant pairs”. | Corrected | Pg. 10;  Line 231 |
|  | Line 206, should read, “Ninety-four (74.6%) of 126 mothers who refused….” | Corrected | Pg. 12;  Line 270 |
|  | Line 218, delete “the cost of” | Deleted |  |
|  | Table 2: The title of the second column is not clear. It is certainly not frequency, but number (n) of individuals affected. The total of 291 can be moved to the title of the table. | Frequency has been changed to number of participants (n)  The total number of participants in each column/row has been indicated | Pg. 13;  Line 291 |
|  | The section on “Reasons for not wanting self/child to be screened” should be in a separate table since the denominator is not 291, but the 126 that did not agree to be screened. It this is true, the percentages given in the table cannot be correct. | Yes, 126 participants did not want their child to be screened. But the question referred to r**easons why both mothers and child refused screening. Not only the infants.** Moreover, **t**he question had multiple responses and participants had opportunity to pick more than one answer. This was indicated by the sign (*) with the explanation given below the table. | Table 2; page 13 |
| **Discussion:** | Apart from the accuracy of HemoTypeSC and its low cost, the other disadvantages of HPLC in a resource-poor environment should be stressed. These include the need for highly skilled personnel, reagents and electrical power, which, is quite often, not available. | This point has been stated in the introduction. To avoid repetition in the discussion. | Pg. 4;  Lines 90-99 |
|  | Line 292 - 293, should read “…with the creation of a comprehensive treatment center in each of the 6 geopolitical zones….” | Corrected | Pg. 16;  Line 355 |
|  | Line 305, what is DHS? | demographic and health survey. Now included | Pg. 17;  Line 368 |
|  | Line 316, should read “The mothers of the two infants who were diagnosed…..” | Corrected | Pg. 17;  Line 379 |
|  | Line 330, a new paragraph should begin with the sentence, “The majority of the mothers…..” | Done. | Pg. 18;  Line 391 |
| **Reviewer #2:** |  |  |  |
|  | Reference is made to a paper reporting that only 36% deliveries take place in health facilities in Nigeria yet 95% of the studied population delivered in hospitals/health centres. | Thank you, sir, for this point.  The 95% in this study **only** attended antenatal clinic in hospital/health facilities but not delivered in same. | Table 1; pg.11 |
|  | It would also be helpful to know what proportion of births attend ‘routine immunization clinics’. | According to Nigerian Health and Demographic Survey (NDHS) 2018 report  Overall, 31% of children have received all basic vaccinations, and 21% have received all age-appropriate vaccinations. Sixty-seven percent of children have received BCG, 65% have received the first dose of pentavalent, and 74% have received polio 1. Fifty percent and 47% of children have received the third doses of the pentavalent and polio vaccines, respectively. Coverage of vaccination against measles is 54%.  Nineteen percent of children in Nigeria have not received any vaccinations. |  |
|  | Although 291 mother/infant ‘units’ were admitted to the study, 41 (14%) refused screening and the principle reason appears to be that they believed that they and their spouses had an AA genotype. Using the Bio-Rad D-10 as a reference method, the performance of the diagnostic kit appears acceptable but the numbers are far too small for reliable assessment with the detection of only one SS, one SC and I CC baby. | The study was actually a pilot study as reflected in the title. Hopefully, we intend to conduct bigger research that will cover more of the LGAs in Lagos State. |  |
|  | Overall 53 mothers were believed to have the sickle cell trait but the fathers of 2 infants with sickle cell disease were believed by the mother to have an AA phenotype. This new information should be presented in the results and not in the discussion, along with the possible explanation – were these beta thalassaemia traits, not the fathers, or assumed to be normal because they were well. | It was 53 babies who were diagnosed to have sickle cell traits and not mothers.  The information is presented in the result. | Table 3; pg.15 |
|  | The difference between ‘willingness’ and ‘affordability’ in Table 2 was not clear to this reviewer. | Willingness in table 2 refers to the readiness of the participants to pay for the screening tests without being persuaded.  While affordability refers to their ability to provide the money required for the screening tests without detriment to the person making payment. | Table 2; pg.13 |
